# Role of Inferior Vena Cava (IVC) Recanalization in Patients with Back Pain, Secondary to IVC Obstruction in Budd–Chiari Syndrome

**DOI:** 10.3390/diagnostics13030438

**Published:** 2023-01-25

**Authors:** Vijay Kubihal, Amar Mukund, Yasha Pandey, Chitranshu Vashistha, Rakhi Maiwall, Yashwant Patidar, Anil Yogendra Yadav, Roshan Lal Koul, Shiv Kumar Sarin

**Affiliations:** 1 Department of Interventional Radiology, Institute of Liver and Biliary Sciences, New Delhi 110070, India; 2 Indira IVF Fertility Center, New Delhi 110008, India; 3 Department of Hepatology, Institute of Liver and Biliary Sciences, New Delhi 110070, India; 4 Department of Anaesthesia, Institute of Liver and Biliary Sciences, New Delhi 110070, India; 5 Department of Neurology, Institute of Liver and Biliary Sciences, New Delhi 110070, India

**Keywords:** Budd–Chiari syndrome, IVC obstruction, back pain, IVC recanalization

## Abstract

Purpose: To study the prevalence of back pain in patients of Budd–Chiari syndrome (BCS) with inferior vena cava (IVC) obstruction, and to evaluate the role of IVC recanalization in resolution of back pain. Methods: All patients with BCS and IVC obstruction who underwent IVC recanalization between January 2018 and October 2022 were included. Patients with degenerative spine disease or other identifiable causes for back pain were excluded; remaining patients were assessed for the presence of back pain. In patients with back pain, pain relief was assessed at 24 h following IVC recanalization. Results: Fifty-eight patients with BCS and IVC occlusion were identified, of which six with degenerative spine diseases were excluded. Of the remaining 52 patients, 34 (65.4%) had back pain, with pain score between 3 and 9. Engorged epidural venous plexus on preprocedural imaging (*p* = 0.002), and degree of luminal narrowing (*p* = 0.021) had a significant association with back pain. Twenty-nine of thirty-four patients (85.3%) with back pain had pain relief immediately following IVC recanalization, more so in patients with engorged epidural venous plexus on preprocedural imaging (*p* < 0.001). Conclusion: Back pain is one of the under-reported symptoms of IVC obstruction in BCS. IVC recanalization by IVC angioplasty with or without stenting relieves back pain due to the decompression of engorged epidural veins.

## 1. Introduction

Budd–Chiari syndrome (BCS) is a rare condition, with incidence ranging from 0.1 to 10 per million population per year, and which is more common in developing countries [1]. It occurs secondary to occlusion of hepatic veins or supra-hepatic inferior vena cava (IVC) or both [2]. Patients with IVC obstruction in BCS often present with hepatic dysfunction, portal hypertension, and lower-limb edema [3,4,5]. There is limited literature on the association between IVC obstruction and back pain [3,6,7,8,9,10,11,12,13,14]. In normal individuals, veins from the spinal cord drain via valveless radicular veins into the vertebral venous plexus, also known as the Batson plexus. The vertebral venous plexus is formed by the internal plexus located in the extradural space, called the epidural venous plexus, the external plexus around the vertebral column, and the basivertebral vein, which passes through the vertebral body. The internal and external venous plexus are linked together by intervertebral veins, through the intervertebral foramen, and drain via segmental vein into the larger extraspinal veins, such as the ascending lumbar vein and azygous vein. In patients with IVC obstruction, blood from the lower extremities, abdomen, and pelvis is routed to the azygous vein via paravertebral veins and the vertebral venous plexus, causing engorgement of the epidural intrinsic vertebral venous plexus (epidural venous plexus enlargement, EVE), which can mimic symptoms of lumbar disc herniation or spinal canal stenosis. Further, increased venous pressure can be transmitted to the spinal veins through valveless radicular veins and cause congestive myelopathy [3,12,15]. Back pain may be the only presentation of IVC obstruction in some patients. Often, patients with back pain are evaluated with a radiograph of the spine and/or MRI which adds to the cost of management. Radiological intervention in the form of IVC recanalization and/or anticoagulation can help restore blood flow through the IVC, decongesting the distended paraspinal and intraspinal veins, and thus relieving back pain in such patients [3,6,7,8,9,10,11,12,13,14]. In our study, we evaluated the prevalence of back pain in patients with IVC obstruction and BCS, and also the role of IVC recanalization in the resolution of back pain.

## 2. Methodology

A retrospective search of hospital databases was conducted for patients with BCS and IVC obstruction, who underwent radiological intervention at ILBS between January 2018 and October 2022, and 58 patients were identified. Radiological interventions included IVC recanalization followed by IVC angioplasty with or without stent placement. All the patients received oral anticoagulation as an adjunct to IVC recanalization. Inclusion and exclusion criteria are mentioned below.

### 2.1. Inclusion Criteria

Patients with BCS and IVC obstruction, who underwent radiological intervention from January 2018 to October 2022.

### 2.2. Exclusion Criteria

#### Patients Lost to Follow Up

Patients with any identifiable cause of back pain were excluded for analysis of back pain relief following IVC recanalization.

**Figure 1:**
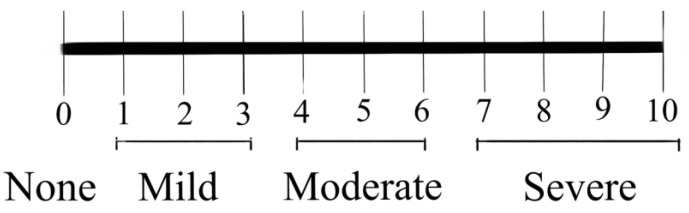
Numerical rating scale for pain assessment.

Clinical features and imaging findings of the patients were analyzed, along with routine laboratory investigations. Contrast-enhanced CT venogram was acquired in all the patients prior to the IVC recanalization. For CT venogram, the patient was positioned supine, with craniocaudal acquisition from the dome of the diaphragm to the ischial tuberosity during inspiration at a delay of 90 to 120 s from the start of IV injection of 100 mL iodinated contrast. Preprocedural imaging (contrast-enhanced CT venogram) was assessed for the level of IVC obstruction (suprahepatic, intrahepatic, or multisegmental), length of IVC obstruction (in mm), and degree of IVC obstruction (complete vs partial), acute vs chronic IVC obstruction, and presence of EVEs (defined as epidural venous plexus of size ≥ 5 mm). Chronic IVC obstruction included patients with chronic stenosis/occlusion, membranous obstruction, caudate lobe hypertrophy, and stent related chronic IVC obstruction, whereas acute IVC obstruction included patients with acute thrombotic occlusion of IVC. IVC recanalization was performed via right internal jugular vein or right femoral vein access, using either balloon catheter of size 18 to 22 mm or wall stent of size 20/22 mm in diameter. Apart from liver function tests, patients were assessed for presence of back pain using a numerical pain-scoring system (Figure 1) at the time of admission and 24 h after radiological intervention, where the patients were asked to rate their back pain on a scale from 0 to 10, where 0 is no pain and 10 is the worst pain possible. A rating of 1 to 3 was taken as mild, 4 to 6 as moderate, and 7 to 10 as severe. Pain relief was defined as improvement in patient’s pain score, with no or mild residual pain (i.e., pain score of less than or equal to 3), without requirement of analgesics.

### 2.3. Statistical Analysis

SPSS 17.0 software was used for data analysis. Qualitative variables are expressed as frequencies, and continuous variables are expressed as mean ± standard deviation (SD) and/or range. Chi-square test or Fisher’s exact test (when cell value is less than 5) were the tests of significance used for categorical data, and Student’s unpaired t-test was used for numerical data. The association of back pain and pain relief (at 24 h following radiological intervention) with age, sex, type of IVC obstruction, length of obstruction, degree of luminal narrowing, and presence of EVEs on preprocedural imaging was assessed. A *p*-value of less than 0.05 was considered statistically significant.

## 3. Results

Of 58 patients included in our study, 38 were male and 20 females, with an age range of 11 to 75 years (mean of 36.9 years). The majority of the patients presented with abdominal distension (*n* = 49), abdominal pain (*n* = 24), and pedal edema (*n* = 28), while jaundice was seen in 13 patients. Improvement in liver function tests were seen in the majority of our patients at 1 month follow-up from IVC recanalization, with a significant decrease in serum bilirubin levels following IVC recanalization (*p* < 0.001).

**Figure 2:**
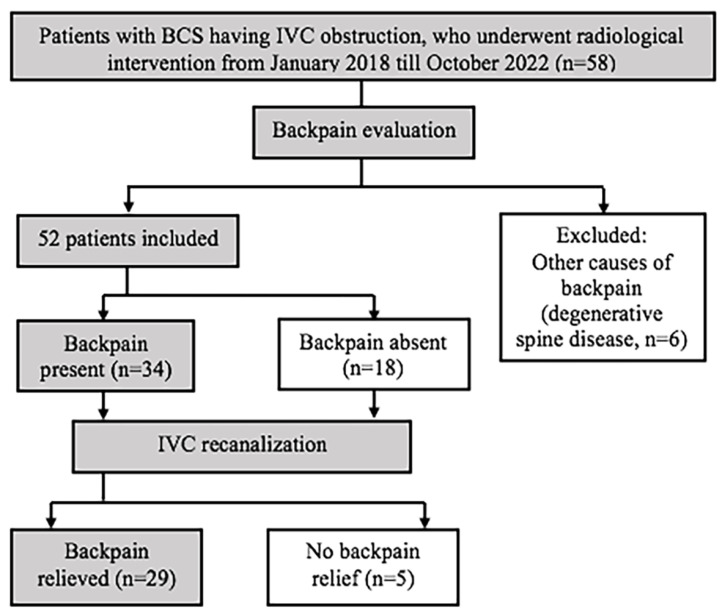
Study design.

Figure 2 shows study design, and Table 1 shows characteristics of study population.

### Evaluation of Back Pain

Out of 58 patients, 6 patients with degenerative spine disease were excluded, and 52 (34 male and 18 female) patients were assessed for the presence of back pain. Out of 52 patients with BCS and IVC obstruction, 34 patients (65.4%) had back pain of varying severity. No patients had other symptoms or signs of myelopathy. Table 2 shows the characteristics of patients with and without back pain.

A significant association was seen between back pain and the presence of EVEs and complete IVC narrowing on preprocedural imaging. No significant association was seen for age, sex, type of IVC obstruction, and length of IVC obstruction. Among 34 patients with back pain, the duration of pain ranged from 5 days to 7 months, with a mean duration of 2 months. The severity of pain, as per the pain score, ranged between 3 and 9, with a mean score of 6. Patients with acute thrombotic obstruction had more severe pain (mean pain score 8.5, standard deviation (SD) 0.7), compared to chronic (mean pain score 5.9, SD 1.4) IVC obstruction (***p* = 0.014**). Patients with complete IVC obstruction had a mean pain score of 6.1 (SD 1.3), whereas patients with partial IVC obstruction had a mean pain score of 6.1 (SD 1.9), and the difference was not statistically significant (*p* = 0.937). Of 34 patients, 29 (85.3%) patients with back pain had pain relief following IVC recanalization, with a mean score of 2.6 at 24 h of intervention. Table 3 shows the characteristics of patients with and without pain relief. The presence of EVEs on preprocedural imaging showed a significant association with pain relief following IVC intervention (*p* < 0.001). No significant association was seen between pain relief and age, sex, type of IVC obstruction, length of IVC obstruction, and degree of luminal narrowing.

**Figure 3:**
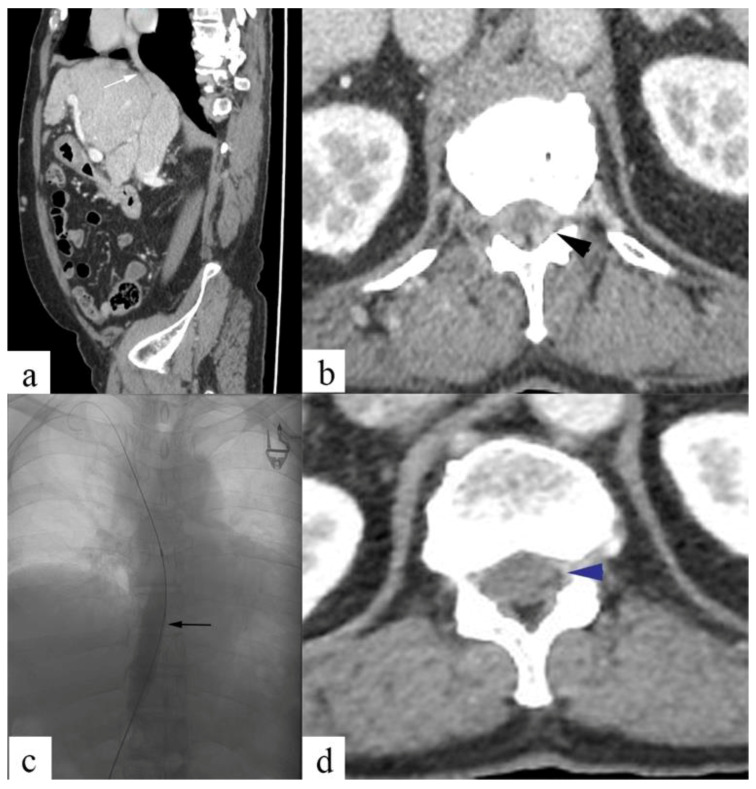
Membranous IVC obstruction in a 29-year-old male patient with Budd–Chiari syndrome and back pain. Contrast-enhanced CT images show membranous IVC obstruction at the suprahepatic IVC (**a**; white arrow) and epidural venous plexus enlargement (**b**; black arrowhead). Balloon angioplasty was performed for IVC obstruction (**c**; black arrow), following which the resolution of EVEs (**d**; blue arrowhead) was noted, along with back pain relief.

Figure 3 shows epidural venous plexus enlargement (EVE) in a 29-year-old male patient with Budd–Chiari syndrome, and back pain and resolution of EVE following IVC angioplasty.

**Figure 4:**
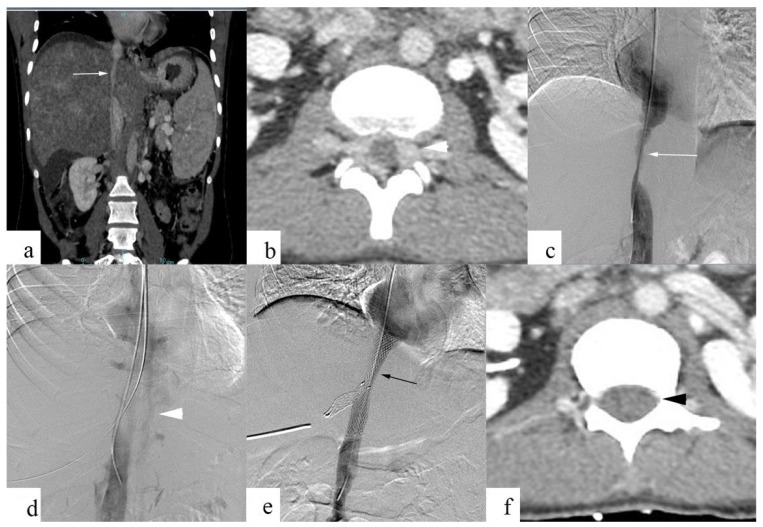
Chronic IVC stenosis in a 32-year-old male patient with Budd–Chiari syndrome and back pain. Contrast-enhanced CT and digital subtraction angiographic images show chronic IVC stenosis involving intrahepatic IVC (**a**,**c**; white arrow), and paravertebral venous plexus and epidural venous plexus enlargement (**b**,**d**; white arrowhead). Balloon angioplasty, primary stenting, and hepatic venous obstruction (**e**; black arrow) were performed for IVC, following which the resolution of EVEs (**f**; black arrowhead) was noted, along with back pain relief.

Figure 4 shows epidural venous plexus enlargement (EVE) in a 32-year-old male patient with Budd–Chiari syndrome, and back pain and resolution of EVE following IVC stenting.

## 4. Discussion

BCS is a rare condition, and often presents along with hepatic dysfunction, portal hypertension, and lower extremity swelling. Back pain in patients with IVC obstruction is under-reported, but can be of significant concern to the patient. There are only case reports or case series which have evaluated back pain in patients with IVC obstruction.

Table 4 shows some of the literature that have reported back pain in patients with IVC obstruction.

These studies show the association of back pain with IVC obstruction, probably related to epidural venous plexus enlargement, causing compression on the spinal cord and/or congestive myelopathy. Many of the above studies report the successful resolution of symptoms following IVC recanalization procedures and/or anticoagulation. In our study, we evaluated the prevalence of back pain secondary to IVC obstruction, and improvement/resolution of back pain following IVC recanalization. Presence of EVEs on preprocedural imaging had a significant association with back pain (*p* = 0.002) suggesting a possible causative association. Back pain was more common in patients with complete IVC obstruction compared to those with partial IVC obstruction (*p* = 0.02). This could be due to higher back-pressure changes proximal to the obstructed IVC, and more severe EVEs in complete IVC obstruction. No significant association was found between back pain, type of IVC obstruction (acute vs chronic), and length of obstruction. Patients with acute thrombotic obstruction had more severe pain scores compared to chronically occluded IVC patients (*p* = 0.014). These findings suggest that the severity of back pain may vary based on the onset and duration of IVC obstruction. In chronic obstruction, the opening up of additional venous collateral pathways may partially decompress the EVEs, and thus be associated with less severe pain compared to acute IVC obstruction. The further severity of pain may also be explained by differential compression on the spinal cord and nerve roots by EVEs. Of 34 patients with back pain, 29 (85.3%) had pain relief following IVC recanalization (angioplasty alone or with stenting). This suggests that restoration of blood flow within the IVC by angioplasty with or without stenting could relieve back pain in patients of BCS with IVC obstruction.

Limitations of the study include (1) the small sample size, (2) the retrospective nature of the study, and (3) single-center study. We only included the patients with IVC obstruction in BCS, and the majority of our patients had suprahepatic or intrahepatic IVC obstruction. Further studies including patients with IVC obstruction without Budd–Chiari diagnosis may be considered to test the uniformity of the results.

## 5. Conclusions

Back pain is an under-reported symptom of IVC obstruction in Budd–Chiari syndrome, possibly related to increased venous back pressure and epidural venous plexus engorgement causing compression over the spinal cord or exiting nerve roots. Radiological interventions such as IVC angioplasty with or without stenting lead to decongestion of the engorged epidural vein and an improvement in back pain.

## Figures and Tables

**Table 1:** Characteristics of study population.

Number of patients (*n*)	58			
Age [range, mean (SD)]	11 years to 75 years, 36.9 years (13.3 years)			
Sex	Male—38 Female—20			
Cause of IVC obstruction	Acute thrombosis—3 Chronic stenosis / obstruction—25 Membranous obstruction—21 Caudate lobe hypertrophy—7 Stent related—2			
Level of IVC obstruction	Suprahepatic—40 Intrahepatic—8 Multisegmental—10			
Length of IVC obstruction [mean (SD)]	22.3 mm (27 mm)			
Degree of luminal narrowing	Complete obstruction—39 Partial obstruction—19			
IVC recanalization	Angioplasty alone—47 Angioplasty with stenting—11			
Laboratory parameters [mean (SD)]		Pre-procedure	1 month post-procedure	*p*-value
	Serum bilirubin	1.9 (1.5) mg/dL	1.2 (0.4) mg/dL	**<0.001**
	AST	156.8 (860.4) U/L	44.9 (101.1) U/L	0.327
	ALT	55.5 (196.7) U/L	35.9 (60.4) U/L	0.470

(SD—standard deviation, IVC—inferior vena cava, AST—aspartate transaminase, ALT—alanine transaminase).

**Table 2:** Characteristics of patients with and without back pain (*n* = 52).

Characteristics	Back Pain	No Back Pain	
Age	Mean (SD)—34.3 (10.6)	Mean (SD)—34.1 (12.4)	*p* = 0.928
Sex	M—20 F—14	M—14 F—4	*p* = 0.227
Type of IVC obstruction	Acute—2 Chronic—32	Acute—1 Chronic—17	*p* = 1.000
Length of IVC obstruction (mean (SD))	Mean (SD)—21 mm (25 mm)	Mean (SD)—23.8 mm (28.5 mm)	*p* = 0.714
Degree of luminal narrowing	Complete—26 Partial—8	Complete—8 Partial—10	***p* = 0.021**
EVEs	Present—30 Absent—4	Present—8 Absent—10	***p* = 0.002**
IVC recanalization	Angioplasty alone—25 Angioplasty + stenting—9	Angioplasty alone—17 Angioplasty + stenting—1	

(SD—standard deviation, IVC—inferior vena cava, EVE—epidural venous plexus enlargement).

**Table 3:** Characteristics of patients with and without pain relief (*n* = 34 patients with back pain).

Characteristics	Pain Relief Present	No Pain Relief	
Age	Mean (SD)—35.2 (11)	Mean (SD)—29.6 (6.9)	*p* = 0.286
Sex	M—18 F—11	M—2 F—3	*p* = 0.627
Type of IVC obstruction	Acute—2 Chronic—27	Acute—0 Chronic—5	*p* = 1.000
Length of IVC obstruction (mean (SD))	Mean (SD)—21.8 mm (26.2 mm)	Mean (SD)—16.1 mm (18.1 mm)	*p* = 0.641
Degree of luminal narrowing	Complete—23 Partial—6	Complete—3 Partial—2	*p* = 0.570
EVEs	Present—28 Absent—1	Present—2 Absent—3	***p* < 0.001**
IVC recanalization	Angioplasty alone—21 Angioplasty + stenting—8	Angioplasty alone—4 Angioplasty + stenting—1	

(SD—standard deviation, IVC—inferior vena cava, EVE—epidural venous plexus enlargement).

**Table 4:** Literature review for back pain in IVC obstruction.

Year	Authors	Type of the Study	Location of IVC Obstruction	Cause	Presenting Features	Intervention	Results
2004	Paksoy and Gormus [6]	Retrospective review of 13 patients	Infrarenal IVC	IVC thrombosis in 10 patients IVC compression by gravid uterus in 2 patients IVC invasion by abdominal malignancy in 1 patient	Intractable low back and radicular pain	Venous thrombectomy in 2 patients with acute IVC thrombosis Anticoagulation in 8 patients with IVC thrombosis Timely delivery in 2 pregnant patients Untreated in patient with inoperable abdominal malignancy	Symptoms resolved in 12 treated patients
2006	Bozkurt et al. [9]	Case report	Hepatic IVC	Budd–Chiari syndrome—caudate lobe hypertrophy	Intractable low back and radicular pain	IVC stent	Symptoms resolved
2006	Mohit et al. [8]	Case report	Renal and Infrarenal IVC	IVC thrombosis	Intractable low back pain Lower extremity numbness and weakness	Mechanical thrombectomy and IVC balloon angioplasty Anticoagulation	Symptoms resolved
2007	Dudeck et al. [7]	Case report	Infrarenal IVC	Agenesis of infraranal IVC DVT of right common iliac vein	Intractable low back and radicular pain	Anticoagulation	Symptoms resolved
2010	Kamerath and Morgan [13]	Case report	Infrarenal IVC	Agenesis of infraranal IVC	Low back and radicular pain Exercise induced numbness in lower extremity Urinary and fecal incontinence	None	Not reported
2014	Tabatabai et al. [11]	Case report	Infrarenal IVC	IVC thrombosis	Intractable low back and radicular pain Lower limb and testicular swelling	Anticoagulation	Significant improvement of symptoms
2015	Lee et al. [3]	Case report	Hepatic IVC	Budd–Chiari syndrome—Chronic obstruction	Intractable low back and radicular pain Lower extremity weakness and voiding difficulty	IVC stent	Symptoms resolved
2015	Carvalho et al. [12]	Case report	Hepatic IVC	Chronic IVC obstruction	Low back pain Bilateral lower extremity weakness Urinary retention	IVC stent	Symptoms resolved
2015	Donmez [14]	Case report	Entire IVC	Thin IVC with absence of left common iliac vein	Intractable back pain	Prophylactic antithrombotic	Not reported

## Data Availability

We would like to inform that the data is available with us (not online or on a public platform), and can be shared with reviewers and editors if and when required.
